# Beyond vertebrates: *Drosophila melanogaster* as a model to study negative symptoms of schizophrenia

**DOI:** 10.3389/fpsyt.2025.1622281

**Published:** 2025-07-23

**Authors:** Maximiliano Elgueta-Reyes, Sergio Hidalgo, Jorge M. Campusano

**Affiliations:** ^1^ Centro Interdisciplinario de Neurociencia UC, Facultad de Ciencias Biológicas, Pontificia Universidad Católica de Chile, Santiago, Chile; ^2^ Facultad de Ciencias Biológicas, Pontificia Universidad Católica de Chile, Santiago, Chile; ^3^ Department of Integrative Physiology and Neuroscience, Washington State University, Pullman, WA, United States

**Keywords:** *Drosophila*, schizophrenia, negative symptom, dysbindin-1 (DTNBP1), Rim1, neuroligin

## Abstract

Schizophrenia is a complex neuropsychiatric disorder characterized by positive, negative, and cognitive symptoms. While positive symptoms have been extensively studied, negative symptoms—such as anhedonia, social withdrawal, and apathy—remain challenging to model and treat. Vertebrate animal models for schizophrenia have provided insights into some of the underlying mechanisms associated with this disorder. Recently, *Drosophila melanogaster* has emerged as a valuable model due to its genetic tractability, conserved neurochemical pathways as compared to vertebrates, and suitability for high-throughput behavioral analyses. Mutations in genes such as *dysb1*, *Rim*, and *Neuroligins* have been linked to behaviors in flies resembling negative symptoms of schizophrenia, supporting the relevance of this animal model in psychiatric research. Moreover, behavioral paradigms aimed at assessing social interaction, motivation, and anhedonia in *Drosophila* are being refined to better capture schizophrenia-related deficits. The use of *Drosophila* enables precise investigation of neural circuits and molecular pathways underlying negative symptoms of schizophrenia, research that has the potential to lead to novel therapeutic targets.

## Introduction

Schizophrenia is a complex and multidimensional neuropsychiatric disorder, affecting approximately 1% of the global population, which exhibits a higher prevalence in males (1–3). Globally, costs associated to schizophrenia are estimated between US$94 and US$102 billion annually. This represents an economic burden equivalent to 0.02% to 1.65% of a country’s gross domestic product (GDP), with indirect costs—such as lost productivity and social security expenses—accounting for 50% to 85% of that total (4, 5). This is relevant as the health, social, and economic burden associated to this disorder is substantial, impacting patients but also families, caregivers and society at large.

**Figure 1:**
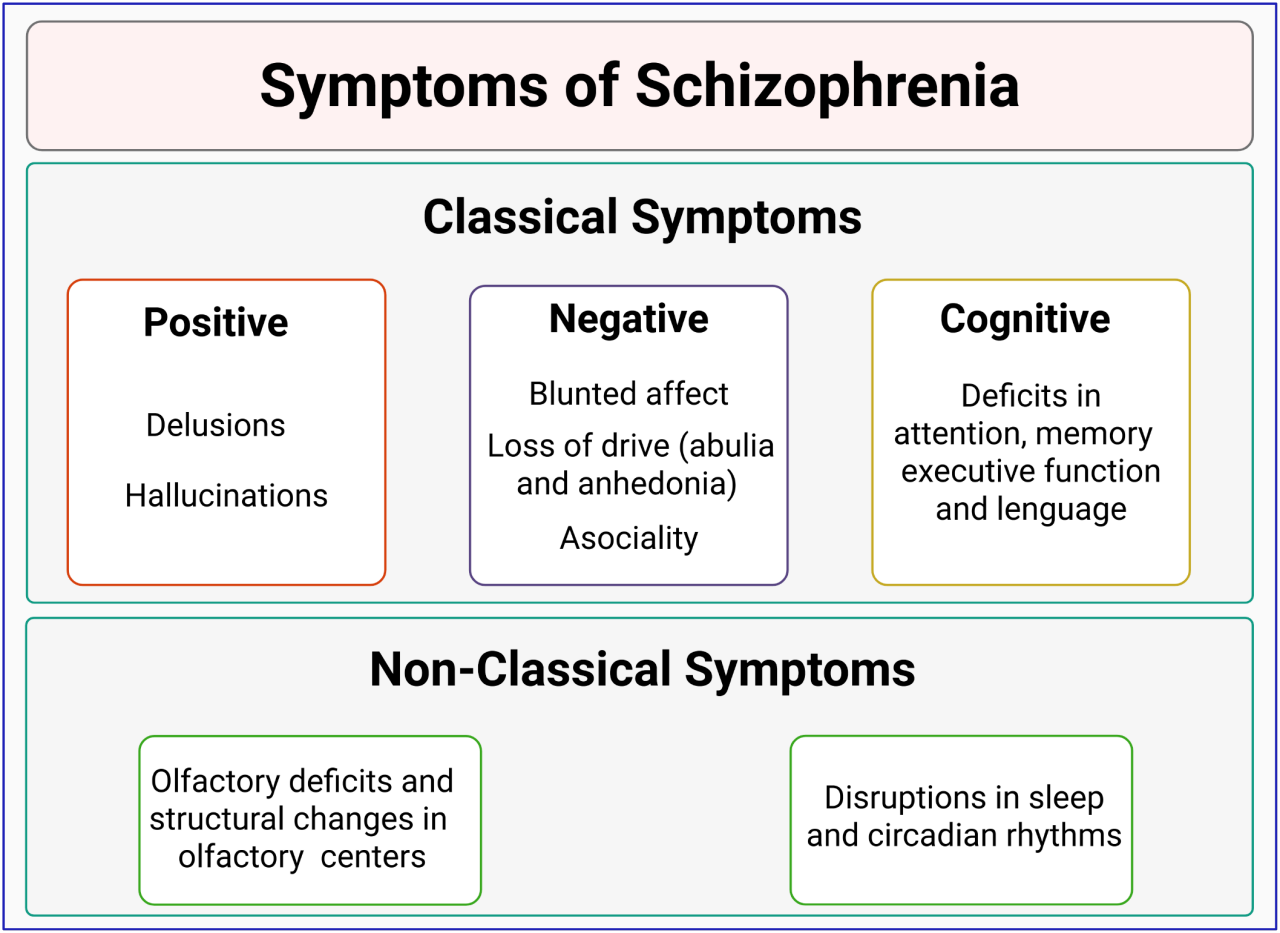
Classification of schizophrenia symptoms. Schizophrenia is characterized by positive symptoms (such as delusions and hallucinations) and negative symptoms (including blunted affect, poverty of speech, and anhedonia). Cognitive impairments, such as deficits in language, memory, and executive function, are also common. Non-classical symptoms, involving olfactory discrimination deficits and sleep/circadian disruptions, are emerging as potential prodromal markers of schizophrenia.

By 1908, Eugen Bleuler first introduced the term “schizophrenia”, describing personality, perception, and cognitive symptoms in a group of patients (6). Schizophrenia was later categorized into positive (hallucinations, delusions) and negative symptoms (blunted affect, avolition, anhedonia, asociality, and alogia) (6–8). It is currently known that schizophrenia also involves cognitive impairment, including alterations in language, executive function, verbal memory, spatial memory, among other features (9, 10) (**Figure 1**).

On the other hand, non-classical symptoms, such as olfactory impairments (11) and circadian disruptions (12), have been observed in 80% of schizophrenia cases (12–14), and have gained attention as prodromal symptoms or markers of this disorder (**Figure 1**).

The study of schizophrenia has largely focused on positive symptoms due to the effectiveness of antipsychotics on them (15, 16). However, although negative symptoms seem critical in determining the loss in the quality of life of people with a diagnosis of schizophrenia, they remain a major therapeutic challenge.

## Negative symptoms of schizophrenia

Negative symptoms involve behavioral features that are absent or undermined in patients. They are classified into two primary domains: abulia/apathy and diminished emotional expression (17, 18). The first domain is understood as deficits in motivation and pleasure. It involves reduced motivation and goal-directed behavior and decreased pleasure when facing positive experiences (17). Thus, this domain includes individual symptoms (or subdomains) of abulia, asociality, and anhedonia (19).

The second domain involves a decrease in the external expression of emotions (blunted affect) and speech (alogia) (18). Blunted affect or affective flattening is linked with diminished quality of life, depressive symptoms, poor social functioning, emotional withdrawal, negative self-evaluation, and suicide ideation (20), while alogia has been associated with cognitive deficits, such as alterations in semantic memory (21).

Importantly, negative symptoms of schizophrenia are little responsive to dopaminergic agents, which are more effective towards positive symptoms of this disorder (22). Thus, there is a need for a better comprehension of the mechanisms underlying the negative symptoms of schizophrenia, in the search for new treatments and therapeutical approaches.

## Schizophrenia etiology and negative symptoms

The etiology of this disorder involves multifactorial elements ranging from genetic features to risk factors in brain development to environmental influences, which accumulate and interact to produce a wide range of symptoms, mainly in adolescence and youth (23, 24).

Several genetic linkage and GWAS studies have tried to identify genes that could play a role in the disorder, and some of these reports have pointed out a genetic contribution to negative symptoms. Thus, for instance, a strong link has been found between negative symptoms of schizophrenia and chromosome 22q11 microdeletions, as well as with alterations in the *NKAIN2* gene, which encodes a protein that interacts with subunits of the sodium/potassium ATPase (25). Additionally, these studies have identified an association between haplotypes of the *DTNBP1* gene (Dystrobrevin Binding Protein 1, also known as Dysbindin-1), and cognitive and negative symptoms of the disorder (26–28). Notably, dysbindin-1 deficiency affects glutamatergic, GABAergic, and dopaminergic neurotransmission (29, 30), some of the neurochemical systems mostly associated with schizophrenia etiology (31). Similarly, haplotypes and polymorphisms in the gene that encodes COMT, an enzyme involved in dopamine metabolism, have been linked to the severity of schizoaffective negative symptoms (32–35).

The serotonergic system plays a well-established role in regulating mood and affect, some of the features associated with negative symptoms of schizophrenia. Considering this, it was proposed that the serotonergic neurochemical system could play a role in these symptoms and early studies supported this idea (36). Accordingly, pharmacological treatments targeting serotonin receptors have been shown to prevent the loss of gray matter typically observed in schizophrenia patients and to improve cognitive and negative symptoms of this disorder (37, 38).

Importantly, most of these studies support that the interaction of genetic and environmental factors during early neurodevelopment contributes to brain vulnerability and predisposition to develop schizophrenia (31, 39). However, what is the contribution of genes and environment, or what are the exact mechanisms responsible for this effect, is an open question that is difficult to study in humans. In this regard, animal models seem better suited to advance on this issue (40).

## Animal models in the study of negative symptoms of schizophrenia

Despite the inherent limitations of studying a complex human disorder like schizophrenia in vertebrate animal models, research in non-human primates, rodents, and zebrafish has provided valuable insights into the cellular, molecular, and circuit-level underpinnings of some behavioral features of schizophrenia (41–43). Positive symptoms, for example, are often modeled through non-verbal indicators such as hyperlocomotion or stereotypy, while negative symptoms are inferred from behaviors like impaired thigmotaxis, reduced exploration, or diminished social interaction (44).

**Figure 2:**
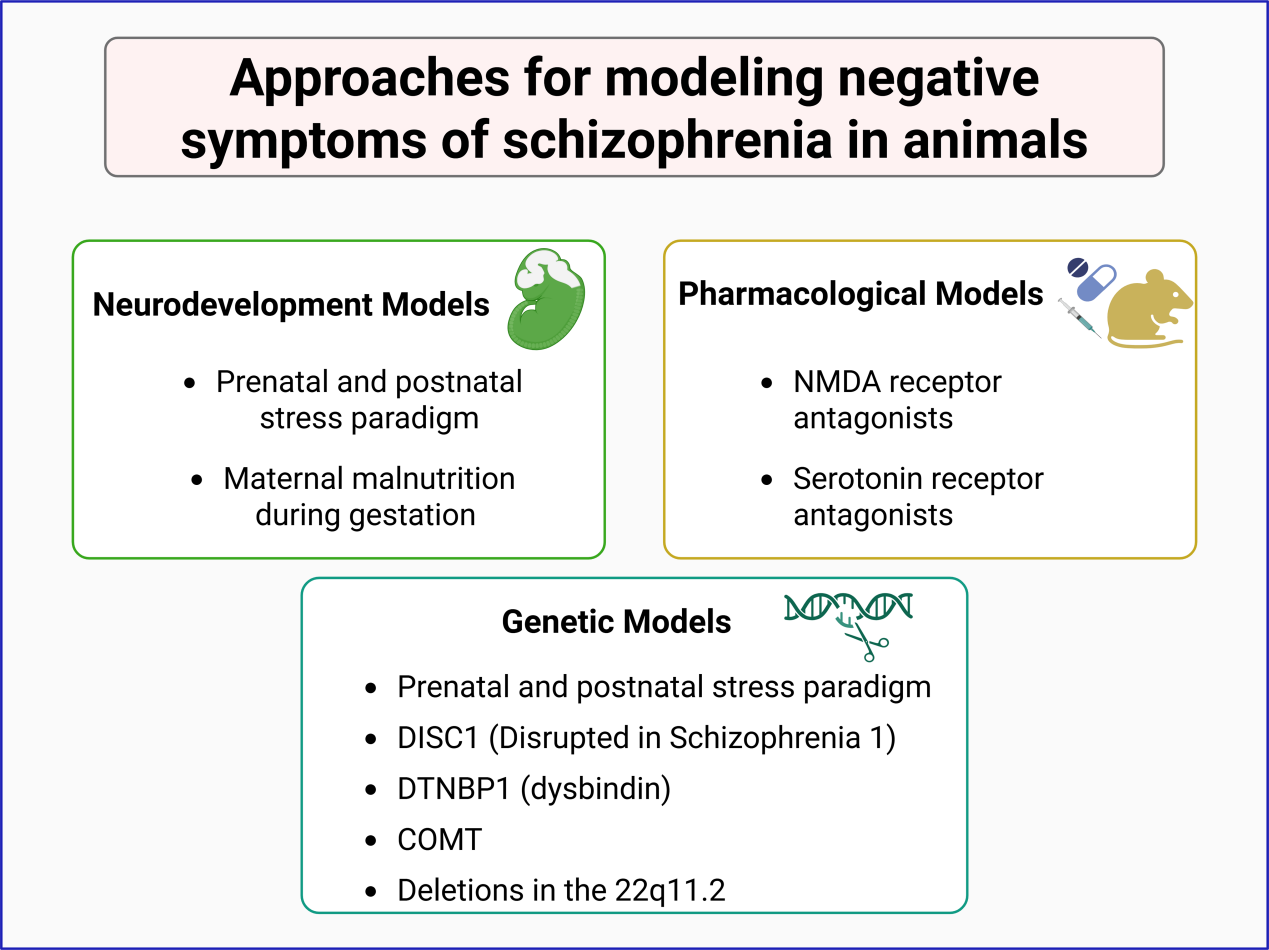
Animal models in the study of negative symptoms of schizophrenia. Experimental models are categorized into three main approaches: neurodevelopmental, pharmacological, and genetic models. Neurodevelopmental models involve prenatal and postnatal stress paradigms or maternal malnutrition during gestation. Pharmacological models utilize NMDA receptor and serotonin receptor antagonists to induce schizophrenia-like phenotypes. Genetic models include mutations in schizophrenia-associated genes such as DISC1, DTNBP1 (dysbindin), COMT, and deletions in the 22q11.2 region. These models contribute to understanding the neurobiological basis of schizophrenia and its negative symptoms.

Rather than replicating the full disorder, animal studies focus on isolating specific symptoms or symptom clusters to explore their underlying causes (45, 46). This strategy has been instrumental in identifying or understanding environmental, genetic, and pharmacological factors contributing to schizophrenia, helping to dissect the complex interplay of elements involved in its pathophysiology (**Figure 2**).

Genetic models in mice to generate schizophrenia-like symptoms include mutants for the DISC1, DTNBP1, and COMT genes (47–50), as well as deletions in the equivalent to 22q11.2 region, among others (51, 52). Most of these tools have been successful in modeling some of the positive symptoms of the disorder.

Environmental models primarily focus on neurodevelopmental disruptions, such as prenatal and postnatal stress paradigms, including maternal exposure to adverse conditions that elevate corticosterone levels, maternal malnutrition during gestation, and maternal separation, resulting in behavioral alterations and schizophrenia-related symptoms in the offspring (53–55).

Pharmacological models offer another widely used approach, employing acute or chronic exposure of animals to specific compounds. For instance, administration of methamphetamine or amphetamine induces hyperlocomotion and stereotypy in rodents, mimicking positive symptoms of schizophrenia and providing support for the dopaminergic hypothesis of this disorder (56, 57). However, these models poorly replicate negative symptoms of the disorder (58, 59). To overcome this limitation, researchers have used NMDA receptor antagonists, such as phencyclidine (PCP) and MK-801, which can induce negative-like symptoms, including social withdrawal, reduced social interaction, and increased immobility in the forced swim test (60–62).

Another pharmacological approach to model negative symptoms of schizophrenia is based on the serotonergic hypothesis for this disorder. This is based, as stated above, on the fact that serotonin dysregulation induces behavioral features that resemble negative symptoms of this disorder and that serotonergic agents show some efficacy against schizophrenia’s negative symptoms (37, 38). Thus, serotonin receptor antagonists, alone or in combination with glutamatergic antagonists or dopaminergic agonists, have been used to generate rodent models for negative symptoms of schizophrenia (63–65) (**Figure 2**).

Although vertebrate models have provided valuable insights into the neurobiological basis of schizophrenia, many of these approaches, particularly pharmacological and neurodevelopmental models, carry the risk of inducing widespread, non-specific alterations in several body organs, multiple neural circuits, and signaling pathways (66–68). Thus, if the goal of these animal models is to understand the contribution of specific circuits or neurochemical systems to the disorder, these global approaches might hinder the precise dissection of the mechanisms contributing to the onset and progression of schizophrenia. Moreover, they might lead to the misidentification of contributing factors. In this regard, the use of models that allow for a more precise spatial and temporal dissection of neural activity and dysfunction is necessary.

## *Drosophila* models for schizophrenia and the study of negative symptoms

In the study of complex human disorders, the use of invertebrate models including *Drosophila melanogaster*, has gained increasing attention. *Drosophila* offers several advantages as an animal model for the study of disorders, including its fully sequenced genome (69), its short life cycle, the possibility to obtain and study a large number of animals, and a high proportion of conserved genes when compared to the human genome (70, 71). Notably, approximately 75% of human disease-related genes have functional orthologs in *Drosophila* (70, 71), including many implicated in schizophrenia. Although exist evident anatomical and structural differences between the brains of flies and vertebrates, the basic principles that govern their development and operation are conserved. Moreover, *Drosophila* connectomics, which has been well established and refined (72, 73), further support using this animal in modeling anatomical and functional features of complex psychiatric and neurodevelopmental disorders like schizophrenia. Furthermore, several binary expression systems have enabled the study of human gene homologs linked to this disorder in *Drosophila* (70, 71). This animal model is particularly valuable because it encompasses the same major neurochemical systems associated with schizophrenia in humans, including dopaminergic, serotonergic, GABAergic, and glutamatergic systems, although some differences in their respective enzymes, receptor subtypes, transporters, and metabolizing proteins need to be considered (74, 75).

Several *Drosophila* models for schizophrenia have been developed and characterized, exhibiting key features observed in other animal models of the disorder and patients. These include altered circadian rhythms, hyperlocomotion, and some cognitive deficits, such as impaired learning and memory (76–80). These models have become valuable tools for exploring the cellular and molecular processes underlying the pathophysiological aspects of schizophrenia, including its negative symptoms, and have provided important information on the human disorder.

Thus, for instance, one of the first studies that explored the molecular underpinnings underlying schizophrenia pathophysiology in *Drosophila* was that of Sawamura et al. (77). In this work, authors generated transgenic flies expressing the human gene *Disrupted in schizophrenia 1*, *DISC1*, which resulted in alterations in sleep homeostasis. Importantly, it was demonstrated that *DISC1* modulates CRE-mediated gene transcription by interacting with ATF4/CREB2 (77), an important factor in a broad range of brain conditions (81–83).

Other studies used *Drosophila* to provide further support to the dopamine ontogenic hypothesis for schizophrenia (76, 84). In these works, activation of the dopaminergic system in specific early developmental windows resulted in behavioral alterations in adult animals, including changes in sleep patterns, and behavioral responses to mechanic and visual stimuli, which could reflect an effect on salience allocation, a characteristic of the positive symptoms of schizophrenia (85).

These and other studies demonstrate the validity of *Drosophila* models to assess the mechanisms underlying complex human disorders including schizophrenia. Nevertheless, one of the challenges in schizophrenia research is the difficulty in replicating negative symptoms in animals -including *Drosophila*- to study the molecular, cellular, and circuital underpinnings underlying their onset. Importantly, new tests and social and cognitive paradigms have been developed over recent years to assess complex behavioral, social, and cognitive functions in *Drosophila* relevant to neurological and psychiatric conditions. For instance, the flies’ clustering behavior, which consists of flies aggregating in groups, has been linked to social coordination and has provided insights into collective behavior dynamics (86). In addition, *Drosophila* exhibits attention-like processes, allowing them to prioritize certain stimuli above others, an aspect of cognition observed in more complex organisms (87). Research has further revealed that *Drosophila* engages in goal-driven behavioral adaptations, modifying their actions based on environmental conditions or experiences, a process akin to motivation, learning, and behavior modifications seen in vertebrates (88). Furthermore, *Drosophila* has been tested in their ability to make choices, which somehow resembles basic decision-making processes (89). These findings highlight the potential of *Drosophila* as a model for studying multifaceted brain processes underlying complex behaviors and foster support that it is possible to study the mechanisms underpinning schizophrenia negative symptoms in this animal.

In this regard, our lab advanced the previous characterization of the hypomorphic mutant *dysb^1^*, which represents a loss-of-function mutation in the fly orthologue of *DTNBP1/Dysbindin-1* (90, 91). Our findings revealed several behavioral phenotypes reminiscent of schizophrenia’s negative symptoms in humans. In particular, *dysb^1^* flies exhibit increased social spacing compared to controls (92). This is in agreement with previous studies in the “sandy” mouse (mutant for dysbindin-1) (93) and in schizophrenia patients, which demonstrate alterations in social distance (94, 95), supporting the notion that social space is a good marker or probe for negative symptoms of schizophrenia.

We further showed neurochemical alterations in the *dysb^1^* mutant flies, including reduced serotonin levels and a two-fold increase in *dSERT* expression (92). Interestingly, the administration of 4-MTA, a serotonin-releasing agent, effectively increased social behaviors in control flies but failed to elicit the same effect in *dysb1* mutants, providing further support for the idea that the serotonergic system plays a role in the expression of negative symptoms of schizophrenia (92).

In a different work (80) we investigated the role of the orthologue for the Rab-3 interacting molecule-1 (*RIM1*) gene, called *Rim* in *Drosophila*, to some of the behavioral anatomical and functional phenotypes observed in schizophrenia patients. In this work, *Rim* mutants displayed impaired social behavior, which is similar to the social impairment described in RIM1α−/− mutant mice (96, 97). Moreover, the *Rim* mutant flies showed impaired olfactory acuity and circadian defects, including a loss of circadian rhythmicity and decreased period length phenotypes, that mapped to the pacemaker ventral lateral clock neurons. Importantly, haloperidol, a typical antipsychotic, efficiently rescued *Rim* mutant deficits to normal levels further validating the *Drosophila* model for investigating the mechanisms underlying schizophrenia-related behaviors (80). Other studies have used *Drosophila* to assess the role that could play alterations in Neuroligins (NLGs) to schizophrenia symptoms. NLGs are a family of proteins that form protein-protein complexes essential for the proper formation, maturation, and functional adjustment of chemical synaptic connections between neurons (98, 99). Several alterations in genes coding for NLGs have been associated with changes in social behavior in disorders such as autism and schizophrenia (100). Specifically, mutations in orthologs for these genes in *Drosophila* (*dlng2* and *dlng4*) have shown alterations in the sleep rhythms, altered acoustic communication signals, as well as a reduced tendency to form groups and social interactions (101–103), phenotypes that parallel negative symptoms observed in schizophrenia.

Recent studies have expanded the scope of *Drosophila* schizophrenia models to study endophenotypes, heritable and quantifiable traits that serve as intermediate markers linking genetic risks to clinical symptoms of a disorder. For instance, Foka et al. (104) demonstrated that *Drosophila furin1* mutants exhibit defective habituation to repeated stimuli, a phenotype that mirrors impaired habituation observed in schizophrenia patients (105). In that work, it was also demonstrated that the deficit observed in flies can be reversed by antipsychotic treatment, validating the translational relevance of this model (104). Likewise, Schiöth et al. (106) provided the first evidence of prepulse inhibition (PPI) for visual stimuli in adult *Drosophila*, an endophenotype sensitive to NMDA receptor antagonists in flies that has been reported in people with this disorder (107).

## Conclusion

*Drosophila melanogaster* has proven to be a valuable model for investigating some of the neurobiological underpinnings of schizophrenia, particularly its negative symptoms, which remain one of the most challenging aspects to study in this disorder. These findings not only affirm the relevance of these genes to the disorder but also underscore *Drosophila* as a model system for investigating the mechanisms involved in psychiatric conditions. Moreover, the development of new behavioral paradigms, such as sucrose preference to assess anhedonia (108), and the forced swim test to measure despair-related behavior (109), further expands the utility of this model. As research continues to refine these approaches, *Drosophila* holds significant potential for deepening our understanding on the cellular and molecular mechanisms driving schizophrenia and for identifying new therapeutic targets to alleviate its debilitating negative symptoms.

## References

[1] Ripke S Neale BM Corvin A Walters JTR Farh K-H Holmans PA. Biological insights from 108 schizophrenia-associated genetic loci. Nature. (2014) 511:421–7. doi: 10.1038/nature13595, PMID: 25056061 PMC4112379

[2] Rossler W Salize HJ van Os J Riecher-Rossler A. Size of burden of schizophrenia and psychotic disorders. Eur Neuropsychopharmacol. (2005) 15:399–409. doi: 10.1016/j.euroneuro.2005.04.009, PMID: 15925493

[3] Tamminga CA Holcomb HH. Phenotype of schizophrenia: a review and formulation. Mol Psychiatry. (2005) 10:27–39. doi: 10.1038/sj.mp.4001563, PMID: 15340352

[4] Evensen S Wisloff T Lystad JU Bull H Ueland T Falkum E. Prevalence, Employment Rate, and Cost of Schizophrenia in a High-Income Welfare Society: A Population-Based Study Using Comprehensive Health and Welfare Registers. Schizophr Bull. (2016) 42:476–83. doi: 10.1093/schbul/sbv141, PMID: 26433216 PMC4753607

[5] Chong HY Teoh SL Wu DB Kotirum S Chiou CF Chaiyakunapruk N. Global economic burden of schizophrenia: a systematic review. Neuropsychiatr Dis Treat. (2016) 12:357–73. doi: 10.2147/NDT.S96649, PMID: 26937191 PMC4762470

[6] Dollfus S Lyne J. Negative symptoms: History of the concept and their position in diagnosis of schizophrenia. Schizophr Res. (2017) 186:3–7. doi: 10.1016/j.schres.2016.06.024, PMID: 27401529

[7] Andreasen NC. Negative Symptoms in Schizophrenia: Definition and Reliability. Arch Gen Psychiatry. (1982) 39:784–8. doi: 10.1001/archpsyc.1982.04290070020005, PMID: 7165477

[8] Crow TJ. The Two-syndrome Concept: Origins and Current Status. Schizophr Bull. (1985) 11:471–88. doi: 10.1093/schbul/11.3.471, PMID: 2863873

[9] Takano H. Cognitive Function and Monoamine Neurotransmission in Schizophrenia: Evidence From Positron Emission Tomography Studies. Front Psychiatry. (2018) 9:228. doi: 10.3389/fpsyt.2018.00228, PMID: 29896132 PMC5987676

[10] Millan MJ Andrieux A Bartzokis G Cadenhead K Dazzan P Fusar-Poli P. Altering the course of schizophrenia: progress and perspectives. Nat Rev Drug Discovery. (2016) 15:485–515. doi: 10.1038/nrd.2016.28, PMID: 26939910

[11] Moberg PJ Turetsky BI. Scent of a disorder: olfactory functioning in schizophrenia. Curr Psychiatry Rep. (2003) 5:311–9. doi: 10.1007/s11920-003-0061-x, PMID: 12857535

[12] Ferrarelli F. Sleep Abnormalities in Schizophrenia: State of the Art and Next Steps. Am J Psychiatry. (2021) 178:903–13. doi: 10.1176/appi.ajp.2020.20070968, PMID: 33726524 PMC8446088

[13] Sakurai T Gamo NJ Hikida T Kim S-H Murai T Tomoda T. Converging models of schizophrenia – Network alterations of prefrontal cortex underlying cognitive impairments. Prog Neurobiol. (2015) 134:178–201. doi: 10.1016/j.pneurobio.2015.09.010, PMID: 26408506 PMC4656070

[14] Zurlo L Dal Bò E Gentili C Cecchetto C. Olfactory dysfunction in schizophrenia and other psychotic disorders: A comprehensive and updated meta-analysis. Schizophr Res. (2025) 275:62–75. doi: 10.1016/j.schres.2024.12.001, PMID: 39671833

[15] Lee J Takeuchi H Fervaha G Sin GL Foussias G Agid O. Subtyping Schizophrenia by Treatment Response: Antipsychotic Development and the Central Role of Positive Symptoms. Can J Psychiatry. (2015) 60:515–22. doi: 10.1177/070674371506001107, PMID: 26720509 PMC4679132

[16] Leucht S Leucht C Huhn M Chaimani A Mavridis D Helfer B. Sixty Years of Placebo-Controlled Antipsychotic Drug Trials in Acute Schizophrenia: Systematic Review, Bayesian Meta-Analysis, and Meta-Regression of Efficacy Predictors. Am J Psychiatry. (2017) 174:927–42. doi: 10.1176/appi.ajp.2017.16121358, PMID: 28541090

[17] Messinger JW Trémeau F Antonius D Mendelsohn E Prudent V Stanford AD. Avolition and expressive deficits capture negative symptom phenomenology: Implications for DSM-5 and schizophrenia research. Clin Psychol Rev. (2011) 31:161–8. doi: 10.1016/j.cpr.2010.09.002, PMID: 20889248 PMC2997909

[18] Kirkpatrick B. Developing concepts in negative symptoms: primary vs secondary and apathy vs expression. J Clin Psychiatry. (2014) 75 Suppl 1:3–7. doi: 10.4088/JCP.13049su1c.01, PMID: 24581452

[19] Ang MS Rekhi G Lee J. Validation of the Brief Negative Symptom Scale and its association with functioning. Schizophr Res. (2019) 208:97–104. doi: 10.1016/j.schres.2019.04.005, PMID: 30987926

[20] Grigoriou M Upthegrove R. Blunted affect and suicide in schizophrenia: A systematic review. Psychiatry Res. (2020) 293:113355. doi: 10.1016/j.psychres.2020.113355, PMID: 32798929

[21] Chang X Zhao W Kang J Xiang S Xie C Corona-Hernández H. Language abnormalities in schizophrenia: binding core symptoms through contemporary empirical evidence. Schizophrenia. (2022) 8:95. doi: 10.1038/s41537-022-00308-x, PMID: 36371445 PMC9653408

[22] Sabe M Kirschner M Kaiser S. Prodopaminergic Drugs for Treating the Negative Symptoms of Schizophrenia: Systematic Review and Meta-analysis of Randomized Controlled Trials. J Clin Psychopharmacol. (2019) 39:658–64. doi: 10.1097/JCP.0000000000001124, PMID: 31688399

[23] Liang SG Greenwood TA. The impact of clinical heterogeneity in schizophrenia on genomic analyses. Schizophr Res. (2015) 161:490–5. doi: 10.1016/j.schres.2014.11.019, PMID: 25496659 PMC4308487

[24] Sawa A Snyder SH. Schizophrenia: diverse approaches to a complex disease. Science. (2002) 296:692–5. doi: 10.1126/science.1070532, PMID: 11976442

[25] Edwards AC Bigdeli TB Docherty AR Bacanu S Lee D de Candia TR. Meta-analysis of Positive and Negative Symptoms Reveals Schizophrenia Modifier Genes. Schizophr Bull. (2015) 42:279–87. doi: 10.1093/schbul/sbv119, PMID: 26316594 PMC4753595

[26] DeRosse P Funke B Burdick KE Lencz T Ekholm JM Kane JM. Dysbindin Genotype and Negative Symptoms in Schizophrenia. Am J Psychiatry. (2006) 163:532–4. doi: 10.1176/appi.ajp.163.3.532, PMID: 16513878

[27] Fanous AH E.J.v.d. Oord BP Aggen SH Neale MC O’Neill FA. Relationship Between a High-Risk Haplotype in the DTNBP1 (Dysbindin) Gene and Clinical Features of Schizophrenia. Am J Psychiatry. (2005) 162:1824–32. doi: 10.1176/appi.ajp.162.10.1824, PMID: 16199828

[28] Wessman J Paunio T Tuulio-Henriksson A Koivisto M Partonen T Suvisaari J. Mixture model clustering of phenotype features reveals evidence for association of DTNBP1 to a specific subtype of schizophrenia. Biol Psychiatry. (2009) 66:990–6. doi: 10.1016/j.biopsych.2009.05.034, PMID: 19782967

[29] Papaleo F Yang F Garcia S Chen J Lu B Crawley JN. Dysbindin-1 modulates prefrontal cortical activity and schizophrenia-like behaviors via dopamine/D2 pathways. Mol Psychiatry. (2012) 17:85–98. doi: 10.1038/mp.2010.106, PMID: 20956979 PMC3388848

[30] Trantham-Davidson H Lavin A. Loss of dysbindin-1 affects GABAergic transmission in the PFC. Psychopharmacology. (2019) 236:3291–300. doi: 10.1007/s00213-019-05285-1, PMID: 31201475 PMC6832803

[31] Howes OD Bukala BR Beck K. Schizophrenia: from neurochemistry to circuits, symptoms and treatments. Nat Rev Neurol. (2024) 20:22–35. doi: 10.1038/s41582-023-00904-0, PMID: 38110704

[32] Molero P Ortuño F Zalacain M Patiño-García A. Clinical involvement of catechol-O-methyltransferase polymorphisms in schizophrenia spectrum disorders: influence on the severity of psychotic symptoms and on the response to neuroleptic treatment. Pharmacogenomics J. (2007) 7:418–26. doi: 10.1038/sj.tpj.6500441, PMID: 17363961

[33] Wang Y Fang Y Shen Y Xu Q. Analysis of association between the catechol-O-methyltransferase (COMT) gene and negative symptoms in chronic schizophrenia. Psychiatry Res. (2010) 179:147–50. doi: 10.1016/j.psychres.2009.03.029, PMID: 20483479

[34] Chen C-Y Lu R-B Yeh Y-W Shih M-C Huang S-Y. Association study of catechol-O-methyltransferase gene polymorphisms with schizophrenia and psychopathological symptoms in Han Chinese. Genes Brain Behav. (2011) 10:316–24. doi: 10.1111/j.1601-183X.2010.00670.x, PMID: 21255265

[35] Pelayo-Terán JM Pérez-Iglesias R Vázquez-Bourgon J Mata I Carrasco-Marín E Vázquez-Barquero JL. Catechol-O-methyltransferase Val158Met polymorphism and negative symptoms after acute antipsychotic treatment in first-episode non-affective psychosis. Psychiatry Res. (2011) 185:286–9. doi: 10.1016/j.psychres.2010.06.006, PMID: 20591499

[36] Spina E De Domenico P Ruello C Longobardo N Gitto C Ancione M. Adjunctive fluoxetine in the treatment of negative symptoms in chronic schizophrenic patients. Int Clin Psychopharmacol. (1994) 9:281–5. doi: 10.1097/00004850-199400940-00007, PMID: 7868850

[37] Meltzer HY. New Trends in the Treatment of Schizophrenia. CNS Neurol Disord Drug Targets. (2017) 16:900–6. doi: 10.2174/1871527316666170728165355, PMID: 28758583

[38] Štrac DŠ Pivac N Mück-Šeler D. The serotonergic system and cognitive function. Trans Neurosci. (2016) 7:35–49. doi: 10.1515/tnsci-2016-0007, PMID: 28123820 PMC5017596

[39] Birnbaum R Weinberger DR. Genetic insights into the neurodevelopmental origins of schizophrenia. Nat Rev Neurosci. (2017) 18:727–40. doi: 10.1038/nrn.2017.125, PMID: 29070826

[40] Damianidou E Mouratidou L Kyrousi C. Research models of neurodevelopmental disorders: The right model in the right place. Front Neurosci. (2022), 16. doi: 10.3389/fnins.2022.1031075, PMID: 36340790 PMC9630472

[41] Blackman RK MacDonald AW Chafee MV. Effects of Ketamine on Context-Processing Performance in Monkeys: A New Animal Model of Cognitive Deficits in Schizophrenia. Neuropsychopharmacology. (2013) 38:2090–100. doi: 10.1038/npp.2013.118, PMID: 23660706 PMC3773669

[42] Demin KA Meshalkina DA Volgin AD Yakovlev OV de Abreu MS Alekseeva PA. Developing zebrafish experimental animal models relevant to schizophrenia. Neurosci Biobehav Rev. (2019) 105:126–33. doi: 10.1016/j.neubiorev.2019.07.017, PMID: 31369798

[43] Langova V Vales K Horka P Horacek J. The Role of Zebrafish and Laboratory Rodents in Schizophrenia Research. Front Psychiatry. (2020), 11. doi: 10.3389/fpsyt.2020.00703, PMID: 33101067 PMC7500259

[44] Tordjman S Drapier D Bonnot O Graignic R Fortes S Cohen D. Animal Models Relevant to Schizophrenia and Autism: Validity and Limitations. Behav Genet. (2007) 37:61–78. doi: 10.1007/s10519-006-9120-5, PMID: 17160702

[45] Yee BK Singer P. A conceptual and practical guide to the behavioural evaluation of animal models of the symptomatology and therapy of schizophrenia. Cell Tissue Res. (2013) 354:221–46. doi: 10.1007/s00441-013-1611-0, PMID: 23579553 PMC3791321

[46] Nani JV Muotri AR Hayashi MAF. Peering into the mind: unraveling schizophrenia’s secrets using models. Mol Psychiatry. (2025) 30:659–78. doi: 10.1038/s41380-024-02728-w, PMID: 39245692

[47] Clapcote SJ Lipina TV Millar JK Mackie S Christie S Ogawa F. Behavioral phenotypes of Disc1 missense mutations in mice. Neuron. (2007) 54:387–402. doi: 10.1016/j.neuron.2007.04.015, PMID: 17481393

[48] Cox MM Tucker AM Tang J Talbot K Richer DC Yeh L. Neurobehavioral abnormalities in the dysbindin-1 mutant, sandy, on a C57BL/6J genetic background. Genes Brain Behav. (2009) 8:390–7. doi: 10.1111/j.1601-183X.2009.00477.x, PMID: 19220483 PMC2774142

[49] Hikida T Jaaro-Peled H Seshadri S Oishi K Hookway C Kong S. Dominant-negative DISC1 transgenic mice display schizophrenia-associated phenotypes detected by measures translatable to humans. Proc Natl Acad Sci. (2007) 104:14501–6. doi: 10.1073/pnas.0704774104, PMID: 17675407 PMC1964873

[50] Pletnikov MV Ayhan Y Nikolskaia O Xu Y Ovanesov MV Huang H. Inducible expression of mutant human DISC1 in mice is associated with brain and behavioral abnormalities reminiscent of schizophrenia. Mol Psychiatry. (2008) 13:173–86. doi: 10.1038/sj.mp.4002079, PMID: 17848917

[51] Managò F Mereu M Mastwal S Mastrogiacomo R Scheggia D Emanuele M. Genetic Disruption of Arc/Arg3.1 in Mice Causes Alterations in Dopamine and Neurobehavioral Phenotypes Related to Schizophrenia. Cell Rep. (2016) 16:2116–28. doi: 10.1016/j.celrep.2016.07.044, PMID: 27524619 PMC5001893

[52] Sumitomo A Horike K Hirai K Butcher N Boot E Sakurai T. A mouse model of 22q11.2 deletions: Molecular and behavioral signatures of Parkinson’s disease and schizophrenia. Sci Adv. (2018) 4:eaar6637. doi: 10.1126/sciadv.aar6637, PMID: 30116778 PMC6093626

[53] Marsden CA King MV Fone KC. Influence of social isolation in the rat on serotonergic function and memory–relevance to models of schizophrenia and the role of 5-HT(6) receptors. Neuropharmacology. (2011) 61:400–7. doi: 10.1016/j.neuropharm.2011.03.003, PMID: 21414329

[54] Meyer U Feldon J. Epidemiology-driven neurodevelopmental animal models of schizophrenia. Prog Neurobiol. (2010) 90:285–326. doi: 10.1016/j.pneurobio.2009.10.018, PMID: 19857543

[55] Weiss IC Feldon J. Environmental animal models for sensorimotor gating deficiencies in schizophrenia: a review. Psychopharmacol (Berl). (2001) 156:305–26. doi: 10.1007/s002130100800, PMID: 11549232

[56] Ceretta APC Schaffer LF de Freitas CM Reinheimer JB Dotto MM Fachinetto R. Gabapentin prevents behavioral changes on the amphetamine-induced animal model of schizophrenia. Schizophr Res. (2016) 175:230–1. doi: 10.1016/j.schres.2016.04.044, PMID: 27179665

[57] Tenn CC Fletcher PJ Kapur S. Amphetamine-sensitized animals show a sensorimotor gating and neurochemical abnormality similar to that of schizophrenia. Schizophr Res. (2003) 64:103–14. doi: 10.1016/S0920-9964(03)00009-4, PMID: 14613675

[58] Cohen-Laroque J Grangier I Perez N Kirschner M Kaiser S Sabé M. Positive and negative symptoms in methamphetamine-induced psychosis compared to schizophrenia: A systematic review and meta-analysis. Schizophr Res. (2024) 267:182–90. doi: 10.1016/j.schres.2024.03.037, PMID: 38554698

[59] Voce A Burns R Castle D Calabria B McKetin R. Is there a discrete negative symptom syndrome in people who use methamphetamine? Compr Psychiatry. (2019) 93:27–32. doi: 10.1016/j.comppsych.2019.06.002, PMID: 31301605

[60] Sams-Dodd F. Phencyclidine in the social interaction test: an animal model of schizophrenia with face and predictive validity. Rev Neurosci. (1999) 10:59–90. doi: 10.1515/REVNEURO.1999.10.1.59, PMID: 10356992

[61] Neill JC Harte MK Haddad PM Lydall ES Dwyer DM. Acute and chronic effects of NMDA receptor antagonists in rodents, relevance to negative symptoms of schizophrenia: a translational link to humans. Eur Neuropsychopharmacol. (2014) 24:822–35. doi: 10.1016/j.euroneuro.2013.09.011, PMID: 24287012

[62] Lim AL Taylor DA Malone DT. Consequences of early life MK-801 administration: long-term behavioural effects and relevance to schizophrenia research. Behav Brain Res. (2012) 227:276–86. doi: 10.1016/j.bbr.2011.10.052, PMID: 22085878

[63] Aghajanian GK Marek GJ. Serotonin model of schizophrenia: emerging role of glutamate mechanisms. Brain Res Rev. (2000) 31:302–12. doi: 10.1016/S0165-0173(99)00046-6, PMID: 10719157

[64] Galici R Boggs JD Miller KL Bonaventure P Atack JR. Effects of SB-269970, a 5-HT7 receptor antagonist, in mouse models predictive of antipsychotic-like activity. Behav Pharmacol. (2008) 19:153–9. doi: 10.1097/FBP.0b013e3282f62d8c, PMID: 18332680

[65] Maxwell J Gleason SD Falcone J Svensson K Balcer OM Li X. Effects of 5-HT7 receptor antagonists on behaviors of mice that detect drugs used in the treatment of anxiety, depression, or schizophrenia. Behav Brain Res. (2019) 359:467–73. doi: 10.1016/j.bbr.2018.11.019, PMID: 30471311

[66] Deutsch SI Mastropaolo J Rosse RB. Neurodevelopmental consequences of early exposure to phencyclidine and related drugs. Clin Neuropharmacol. (1998) 21:320–32., PMID: 9844787

[67] Feifel D Shilling PD. Promise and pitfalls of animal models of schizophrenia. Curr Psychiatry Rep. (2010) 12:327–34. doi: 10.1007/s11920-010-0122-x, PMID: 20544314 PMC2895894

[68] Winship IR Dursun SM Baker GB Balista PA Kandratavicius L Maia-de-Oliveira JP. An overview of animal models related to schizophrenia. Can J Psychiatry. (2019) 64:5–17. doi: 10.1177/0706743718773728, PMID: 29742910 PMC6364139

[69] Adams MD Celniker SE Holt RA Evans CA Gocayne JD Amanatides PG. The genome sequence of Drosophila melanogaster. Science. (2000) 287:2185–95. doi: 10.1126/science.287.5461.2185, PMID: 10731132

[70] Jennings BH. Drosophila – a versatile model in biology & medicine. Materials Today. (2011) 14:190–5. doi: 10.1016/S1369-7021(11)70113-4

[71] Victor Atoki A Maduabuchi AP Salihu ST Nyakundi OE Ismahil AA Victor FI. Exploring the versatility of Drosophila melanogaster as a model organism in biomedical research: a comprehensive review. Fly. (2025) 19:2420453. doi: 10.1080/19336934.2024.2420453, PMID: 39722550 PMC11702942

[72] Dorkenwald S Matsliah A Sterling AR Schlegel P Yu S-c McKellar CE. Neuronal wiring diagram of an adult brain. Nature. (2024) 8032):124–38:634. doi: 10.1038/s41586-024-07558-y, PMID: 39358518 PMC11446842

[73] Schlegel P Yin Y Bates AS Dorkenwald S Eichler K Brooks P. Whole-brain annotation and multi-connectome cell typing of Drosophila. Nature. (2024) 634:139–52. doi: 10.1038/s41586-024-07686-5, PMID: 39358521 PMC11446831

[74] Carvajal-Oliveros A Campusano JM. Studying the contribution of serotonin to neurodevelopmental disorders. Can This Fly? Front Behav Neurosci. (2020) 14:601449. doi: 10.3389/fnbeh.2020.601449, PMID: 33510625 PMC7835640

[75] Yoshihara M Ensminger AW Littleton JT. Neurobiology and the Drosophila genome. Funct Integr Genomics. (2001) 1:235–40. doi: 10.1007/s101420000029, PMID: 11793242

[76] Calcagno B Eyles D van Alphen B van Swinderen B. Transient activation of dopaminergic neurons during development modulates visual responsiveness, locomotion and brain activity in a dopamine ontogeny model of schizophrenia. Transl Psychiatry. (2013) 3:e206. doi: 10.1038/tp.2012.139, PMID: 23299394 PMC3567203

[77] Sawamura N Ando T Maruyama Y Fujimuro M Mochizuki H Honjo K. Nuclear DISC1 regulates CRE-mediated gene transcription and sleep homeostasis in the fruit fly. Mol Psychiatry. (2008) 13:1138–48, 1069. doi: 10.1038/mp.2008.101, PMID: 18762802 PMC2727926

[78] Shao L Shuai Y Wang J Feng S Lu B Li Z. Schizophrenia susceptibility gene dysbindin regulates glutamatergic and dopaminergic functions via distinctive mechanisms in Drosophila. Proc Natl Acad Sci USA. (2011) 108:18831–6. doi: 10.1073/pnas.1114569108, PMID: 22049342 PMC3219129

[79] Hidalgo S Campusano JM Hodge JJL. The Drosophila ortholog of the schizophrenia-associated CACNA1A and CACNA1B voltage-gated calcium channels regulate memory, sleep and circadian rhythms. Neurobiol Dis. (2021) 155:105394. doi: 10.1016/j.nbd.2021.105394, PMID: 34015490

[80] Hidalgo S Campusano JM Hodge JJL. Assessing olfactory, memory, social and circadian phenotypes associated with schizophrenia in a genetic model based on Rim. Transl Psychiatry. (2021) 11:292. doi: 10.1038/s41398-021-01418-3, PMID: 34001859 PMC8128896

[81] Chen L Tang J Liu XQ Li QQ Li JY Li YY. TIGAR Suppresses ER Stress-Induced Neuronal Injury through Targeting ATF4 Signaling in Cerebral Ischemia/Reperfusion. J Neurosci. (2025) 45. doi: 10.1523/JNEUROSCI.1406-24.2025, PMID: 39919831 PMC11949484

[82] Goswami P Akhter J Mangla A Suramya S Jindal G Ahmad S. Downregulation of ATF-4 attenuates the endoplasmic reticulum stress-mediated neuroinflammation and cognitive impairment in experimentally induced alzheimer's disease model. Mol Neurobiol. (2024) 61:5071–82. doi: 10.1007/s12035-023-03861-3, PMID: 38159199

[83] Mamdani F Alda M Grof P Young LT Rouleau G Turecki G. Lithium response and genetic variation in the CREB family of genes. Am J Med Genet B Neuropsychiatr Genet. (2008) 147b. doi: 10.1002/ajmg.b.v147b:4, PMID: 18189280 PMC3549998

[84] Ferguson L Petty A Rohrscheib C Troup M Kirszenblat L Eyles DW. Transient dysregulation of dopamine signaling in a developing drosophila arousal circuit permanently impairs behavioral responsiveness in adults. Front Psychiatry. (2017) 8:22. doi: 10.3389/fpsyt.2017.00022, PMID: 28243212 PMC5304146

[85] Kapur S. Psychosis as a state of aberrant salience: a framework linking biology, phenomenology, and pharmacology in schizophrenia. Am J Psychiatry. (2003) 160:13–23. doi: 10.1176/appi.ajp.160.1.13, PMID: 12505794

[86] Simon AF Chou MT Salazar ED Nicholson T Saini N Metchev S. A simple assay to study social behavior in Drosophila: measurement of social space within a group. Genes Brain Behav. (2012) 11:243–52. doi: 10.1111/j.1601-183X.2011.00740.x, PMID: 22010812 PMC3268943

[87] van Swinderen B Flores KA. Attention-like processes underlying optomotor performance in a Drosophila choice maze. Dev Neurobiol. (2007) 67:129–45. doi: 10.1002/dneu.20334, PMID: 17443778

[88] Pick S Strauss R. Goal-driven behavioral adaptations in gap-climbing Drosophila. Curr Biol. (2005) 15:1473–8. doi: 10.1016/j.cub.2005.07.022, PMID: 16111941

[89] Zhang K Guo JZ Peng Y Xi W Guo A. Dopamine-mushroom body circuit regulates saliency-based decision-making in Drosophila. Science. (2007) 316:1901–4. doi: 10.1126/science.1137357, PMID: 17600217

[90] Dickman DK Davis GW. The schizophrenia susceptibility gene dysbindin controls synaptic homeostasis. Science. (2009) 326:1127–30. doi: 10.1126/science.1179685, PMID: 19965435 PMC3063306

[91] Mullin AP Sadanandappa MK Ma W Dickman DK VijayRaghavan K Ramaswami M. Gene dosage in the dysbindin schizophrenia susceptibility network differentially affect synaptic function and plasticity. J Neurosci. (2015) 35:325–38. doi: 10.1523/JNEUROSCI.3542-14.2015, PMID: 25568125 PMC4287151

[92] Hidalgo S Castro C Zarate RV Valderrama BP Hodge JJL Campusano JM. The behavioral and neurochemical characterization of a Drosophila dysbindin mutant supports the contribution of serotonin to schizophrenia negative symptoms. Neurochem Int. (2020) 138:104753. doi: 10.1016/j.neuint.2020.104753, PMID: 32416114

[93] Hattori S Murotani T Matsuzaki S Ishizuka T Kumamoto N Takeda M. Behavioral abnormalities and dopamine reductions in sdy mutant mice with a deletion in Dtnbp1, a susceptibility gene for schizophrenia. Biochem Biophys Res Commun. (2008) 373:298–302. doi: 10.1016/j.bbrc.2008.06.016, PMID: 18555792

[94] Deus V Jokic-Begic N. Personal space in schizophrenic patients. Psychiatr Danub. (2006) 18:150–8., PMID: 17099605

[95] Di Cosmo G Costantini M Salone A Martinotti G Di Iorio G Di Giannantonio M. Peripersonal space boundary in schizotypy and schizophrenia. Schizophr Res. (2018) 197:589–90. doi: 10.1016/j.schres.2017.12.003, PMID: 29269210

[96] Blundell J Kaeser PS Südhof TC Powell CM. RIM1α and interacting proteins involved in presynaptic plasticity mediate prepulse inhibition and additional behaviors linked to schizophrenia. J Neurosci. (2010) 30:5326–33. doi: 10.1523/JNEUROSCI.0328-10.2010, PMID: 20392954 PMC2860606

[97] Haws ME Kaeser PS Jarvis DL Südhof TC Powell CM. Region-specific deletions of RIM1 reproduce a subset of global RIM1α–/– phenotypes. Genes Brain Behav. (2012) 11:201–13. doi: 10.1111/j.1601-183X.2011.00755.x, PMID: 22103334 PMC3268893

[98] Chubykin AA Atasoy D Etherton MR Brose N Kavalali ET Gibson JR. Activity-dependent validation of excitatory versus inhibitory synapses by Neuroligin-1 versus Neuroligin-2. Neuron. (2007) 54:919–31. doi: 10.1016/j.neuron.2007.05.029, PMID: 17582332 PMC3738748

[99] Varoqueaux F Aramuni G Rawson RL Mohrmann R Missler M Gottmann K. Neuroligins determine synapse maturation and function. Neuron. (2006) 51:741–54. doi: 10.1016/j.neuron.2006.09.003, PMID: 16982420

[100] Kenny EM Cormican P Furlong S Heron E Kenny G Fahey C. Excess of rare novel loss-of-function variants in synaptic genes in schizophrenia and autism spectrum disorders. Mol Psychiatry. (2014) 19:872–9. doi: 10.1038/mp.2013.127, PMID: 24126926

[101] Corthals K Heukamp AS Kossen R Großhennig I Hahn N Gras H. Neuroligins Nlg2 and Nlg4 Affect Social Behavior in Drosophila melanogaster. Front Psychiatry. (2017) 8. doi: 10.3389/fpsyt.2017.00113, PMID: 28740469 PMC5502276

[102] Hahn N Geurten B Gurvich A Piepenbrock D Kästner A Zanini D. Monogenic heritable autism gene neuroligin impacts Drosophila social behaviour. Behav Brain Res. (2013) 252:450–7. doi: 10.1016/j.bbr.2013.06.020, PMID: 23792025

[103] Li Y Zhou Z Zhang X Tong H Li P Zhang ZC. Drosophila neuroligin 4 regulates sleep through modulating GABA transmission. J Neurosci. (2013) 33:15545–54. doi: 10.1523/JNEUROSCI.0819-13.2013, PMID: 24068821 PMC6618453

[104] Foka K Georganta EM Semelidou O Skoulakis EMC. Loss of the schizophrenia-linked furin protein from drosophila mushroom body neurons results in antipsychotic-reversible habituation deficits. J Neurosci. (2022) 42:7496–511. doi: 10.1523/JNEUROSCI.1055-22.2022, PMID: 36028314 PMC9525163

[105] Williams LE Blackford JU Luksik A Gauthier I Heckers S. Reduced habituation in patients with schizophrenia. Schizophr Res. (2013) 151:124–32. doi: 10.1016/j.schres.2013.10.017, PMID: 24200419 PMC3908315

[106] Schioth HB Donzelli L Arvidsson N Williams MJ Moulin TC. Evidence for prepulse inhibition of visually evoked motor response in drosophila melanogaster. Biol (Basel). (2023) 12:635. doi: 10.3390/biology12040635, PMID: 37106835 PMC10135638

[107] Swerdlow NR Talledo J Sutherland AN Nagy D Shoemaker JM. antipsychotic effects on prepulse inhibition in normal ‘low gating’ humans and rats. Neuropsychopharmacology. (2006) 31:2011–21. doi: 10.1038/sj.npp.1301043, PMID: 16482083

[108] Ries A-S Hermanns T Poeck B Strauss R. Serotonin modulates a depression-like state in Drosophila responsive to lithium treatment. Nat Commun. (2017) 8:15738. doi: 10.1038/ncomms15738, PMID: 28585544 PMC5467214

[109] Neckameyer WS A. and R. Nieto-Romero, Response to stress in Drosophila is mediated by gender, age and stress paradigm. (Stress 2015) 18(2):254–66. doi: 10.3109/10253890.2015.1017465, PMID: 25783197 PMC4838016

